# Simulating Next-Generation Sequencing Datasets from Empirical Mutation and Sequencing Models

**DOI:** 10.1371/journal.pone.0167047

**Published:** 2016-11-28

**Authors:** Zachary D. Stephens, Matthew E. Hudson, Liudmila S. Mainzer, Morgan Taschuk, Matthew R. Weber, Ravishankar K. Iyer

**Affiliations:** 1 Department of Electrical and Computer Engineering, Univ. of Illinois at Urbana-Champaign, Urbana, IL, United States of America; 2 Department of Crop Sciences, Univ. of Illinois at Urbana-Champaign, Urbana, IL, United States of America; 3 Institute for Genomic Biology, Univ. of Illinois at Urbana-Champaign, Urbana, IL, United States of America; 4 National Center for Supercomputing Applications, Univ. of Illinois at Urbana-Champaign, Urbana, IL, United States of America; 5 Ontario Institute for Cancer Research, Toronto, ON, Canada; Hospital for Sick Children, CANADA

## Abstract

An obstacle to validating and benchmarking methods for genome analysis is that there are few reference datasets available for which the “ground truth” about the mutational landscape of the sample genome is known and fully validated. Additionally, the free and public availability of real human genome datasets is incompatible with the preservation of donor privacy. In order to better analyze and understand genomic data, we need test datasets that model all variants, reflecting known biology as well as sequencing artifacts. Read simulators can fulfill this requirement, but are often criticized for limited resemblance to true data and overall inflexibility. We present NEAT (NExt-generation sequencing Analysis Toolkit), a set of tools that not only includes an easy-to-use read simulator, but also scripts to facilitate variant comparison and tool evaluation. NEAT has a wide variety of tunable parameters which can be set manually on the default model or parameterized using real datasets. The software is freely available at github.com/zstephens/neat-genreads.

## Introduction

The use of high-throughput sequencing technologies for analyzing genomes has led to an unprecedented increase in the computational complexity of genomic data analysis. In medicine, for example, routine analysis of genomes for individualized clinical treatments is widely anticipated. The analysis complexity is increased in cancer analysis by somatic changes and clonal sub-populations within tumors. Research and medical treatment decisions can be greatly facilitated by use of accurate and rapid variant detection and interpretation software. However, the development of such software is hindered by limited access to real, high quality, high-depth sequencing data for a range of patient and disease phenotypes, and by the lack of “ground truth” information about the variants present in the tissue of origin. Sequencing data from model organisms can be used in some cases, but ultimately they are not fully predictive for humans [1]. In contrast, simulated datasets can be constructed to mimic many properties of human data while also being freely shareable among software developers without exposing personal health information. Thus, simulations can provide a gold standard available to all software engineers for the design and evaluation of variant calling workflows. Synthetic data are functionally similar to the output of a sequencer, but all of the underlying mutational events are known.

There are a number of existing software packages available for generating synthetic NGS read data, each tending to specialize on a particular attribute of a dataset. For example, ART [2], CuReSim [3], GemSim [4], and pIRS [5] focus on realistically emulating the biases inherent in the base calling of various next-generation sequencing (NGS) platforms. Other simulators seek to incorporate more sophisticated models for GC-content biases and copy number variation [6]. None of these simulators, however, offer the ability to easily sweep over the parameters that adequately describe an NGS dataset. For this reason we developed our own software package, the NExt-generation sequencing Analysis Toolkit (NEAT). NEAT is designed to be more flexible and user-friendly than many others in the field (Table 1). The list of existing simulators compared against are those most often used, according to number of paper citations: ART, CureSim, dwgsim [7], GemSim (including the the targeted sequencing functionality of Wessim [8]), Mason [9], pIRS, and SInC [6]. VarSim [10] is not explicitly listed as it is a wrapper around DWGSIM and ART.

**Table 1:** Comparison of read simulator features.

		ART	CuReSim	dwgsim	Gemsim	Mason	pIRS	SInC	NEAT
Mutation models	SNPs / indels		×	×	×		×	×	×
	Structural variation						*	×	*
	Any ploidy						*		×
	Learnable from data				*				×
	Accepts input variants			*	*				×
Sequencing models	Learn Q-score profile	×	*		×	*	×	×	×
	Learn error statistics	×	*		×	*	×	×	×
	GC% coverage bias		×		×		×		×
	Learn fragment lengths				*				×
Usability	Any read length	×	×	×	×		*	×	×
	Single & paired ended reads	×		×	×			×	×
	Any error rate	×	×	×			×		×
	Any mutation rate	×		×	×		×	×	×
	Targeted sequencing			×	×				*
Ground truth	Mapping positions	×	×	×		×			×
	CIGAR alignment	×				×			×
	Variant positions			×			×	*	×

Comparison of the main features of several existing read simulator packages.

×: feature is present in the simulator.

*: feature is either partially implemented or requires significant effort to fully use.

### NEAT Read Simulator

The goal of NEAT is to give users complete control over as many parameters of sequencing data as possible. The objective is not to model or simulate biological or sequencing processes, but rather to faithfully reproduce the properties of sequencing data themselves. In other words, given a particular set of FASTQs or BAMs from any individual or sequencing platform, the user should be able to reproduce the statistical properties of that original dataset in simulation, without directly copying the original variants.

NEAT is a toolkit, providing not only the simulator software to generate reads, but also a set of scripts to extract many of the simulation parameters from real data. It also produces “golden” BAM and VCF files containing the ground truth read alignments and variant locations, which can be used to assess the accuracy of bioinformatics workflows. The software is flexible enough to simulate, in a controlled fashion, the typical sets of mutations, genome ploidy, and clonality of the sampled cell population, and the characteristics of the sequencing platform (read length, error rates, biases in the error types) used to generate the data. We believe this to be the minimum required functionality for a good, generic simulator. In addition, NEAT has been designed to be extensible for any future mutation models, sequencing technologies and sampling procedures. NEAT’s ease of use surpasses existing tools because simulation of an arbitrary NGS dataset can be accomplished in a single command. NEAT is written in Python 2.7 and requires NumPy [11].

## Methods

NEAT is more flexible than existing tools due to its ability to use custom *mutation models* and *sequencing models*. The sequencing models are derived from real sequencing data to mimic the errors and artifacts of DNA sequencing processes. The mutation models are also derived from real data to emulate the distribution of variants in the sample, with a particular emphasis on cancer. The user can select among default models, or derive their own with the included scripts. Contributing to the flexibility of NEAT, the user is able to control several key attributes of an NGS dataset: choice of single-ended or paired-ended reads, read length, average error rate and average mutation rate, regardless of the mutation and sequencing models selected. The quality score profiles can be scaled to arbitrary simulated read length, regardless of the length of reads used to derive the model. Similarly, the frequencies of inserted mutations and sequencing errors can be re-scaled to user-defined values.

**Fig 1:**
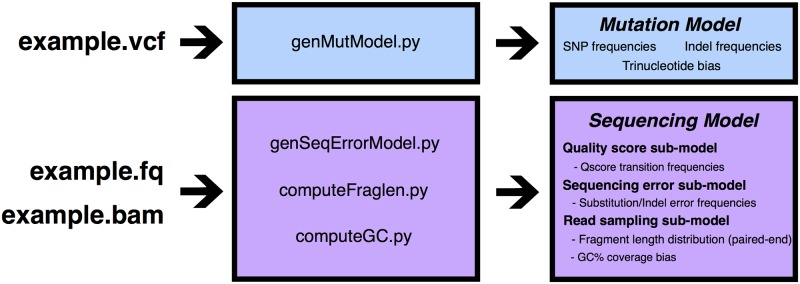
Overview of mutation and sequencing model generation.

**Fig 2:**
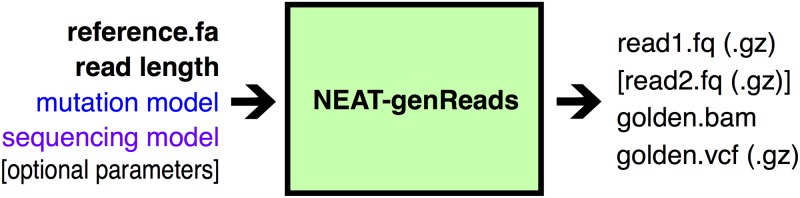
Overview of NEAT Read Simulator.

NEAT takes three mandatory inputs: (1) a reference genome sequence from which to sample reads, (2) read length, and (3) output file name prefix (Figs 1 and 2). The user may also supply a list of specific regions from which to sample predominantly (e.g., to simulate part of a chromosome or restrict to the exome). NEAT can accept an input VCF file containing mutations to insert, in addition to randomly generated mutations. The program outputs simulated FASTQ files as well as “golden” SAM/BAM and VCF files containing the ground truth mapping and variant information, including genotypes.

### Mutation Model Description

From variant call data (e.g. VCF or TSV files) we derive a *mutation model*: probabilities that NEAT uses to insert mutations into the simulated dataset. This mutation model captures single nucleotide substitution (SNP) and indel mutation rates, indel length distributions, as well as substitution base transition probabilities as a function of the nucleotide at that position, its trinucleotide context, and the reference positional context, such as intron, exon, CDS or intergenic region.

Probabilities are captured by region in the mutation model and include the following:

1. *P*(any mutation occurs ∣ genomic position)
2. *P*(substitution ∣ mutation occurs), *P*(insertion ∣ mutation occurs), *P*(deletion ∣ mutation occurs)
3. *P*(substituted base = *Y* ∣ trinucleotide context = *X*_*Z*)
4. *P*(length = *L* ∣ insertion occurs), *P*(length = *L* ∣ deletion occurs)

By default, NEAT introduces all mutations with equal probability. This option can be useful when testing variant calling software in order to have a simple, baseline simulated dataset. However, when mutation models are specified, NEAT will produce more realistic data by sampling from those distributions.

NEAT inserts mutations by working through the reference in sliding windows. If the user has provided an optional BED file of positional mutation rates (a), then the BED regions affecting the current window will be used to construct a distribution *P*(*n* = variant position), where the probability of selecting position *n* to insert a mutation is proportional to the user-specified mutation rate at that position. If no such BED file is provided, the mutations will be inserted across the window such that the total number of mutations is determined by multiplying the length of the window by the desired overall mutation rate. Next, we sample from the mutation models (b) to determine if the mutation should be a SNP, insertion, or deletion. If the mutation should be a substitution, we examine its surrounding nucleotides: they determine our selection of the base transition matrix (c). Then we sample the new nucleotide that will replace that position. Because trinucleotides are not distributed evenly across the genome, care is taken to encode their distribution probabilities correctly by indexing the reference with respect to the trinucleotide distribution. If the mutation should be an insertion or deletion, we sample its length from the learned distribution (d) and alter the affected reference nucleotides. In the current version of NEAT, large-scale structural variation is not introduced by default, but only if the user specifies the variants via an input VCF file.

Mutations with arbitrary variant allele frequency are simulated by generating multiple copies of the reference genome and inserting mutations into a specified fraction of the copies. For example, when simulating a tetraploid, a variant with genotype “0 ∣ 1 ∣ 0 ∣ 0” results in 4 copies of the reference sequence, the second of which is altered to include the inserted mutation. The default simulated ploidy is 2.

### Example Mutation Models

**Fig 3:**
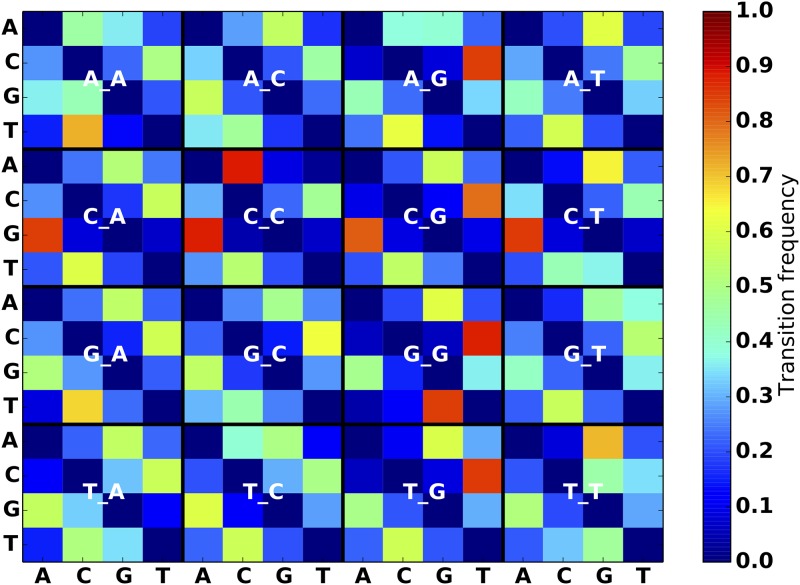
SNP substitution frequency matrices for breast cancer model. The label for each 4 × 4 matrix specifies the nucleotide immediately preceding and following the SNP position. For example, row 3 column 2 of the “A_A” matrix specifies the frequency of AGA mutating into ACA, as observed in the breast cancer SSM dataset.

**Fig 4:**
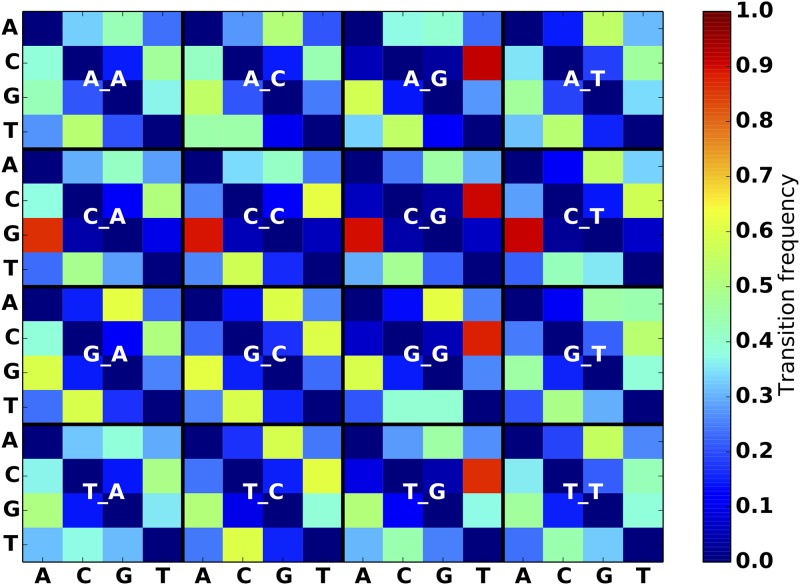
SNP substitution frequency matrices for Leukemia model.

**Fig 5:**
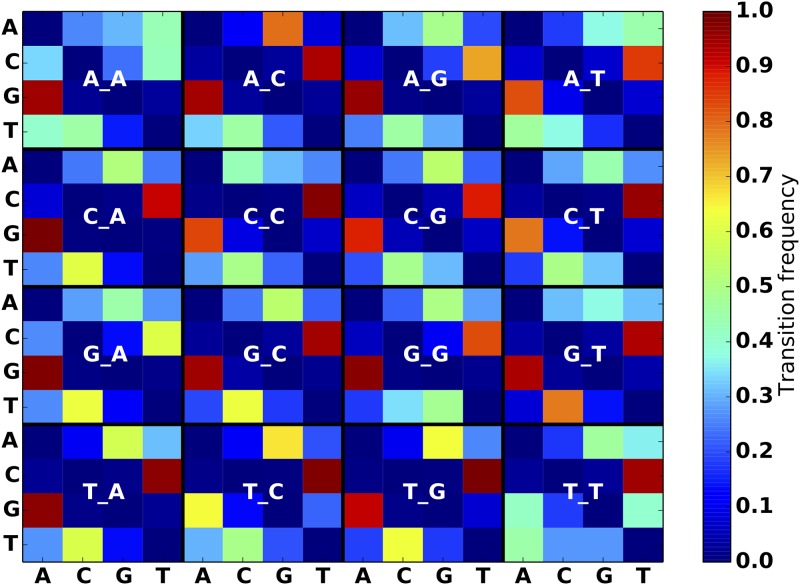
SNP substitution frequency matrices for Melanoma model. Note the strong preference for G → A and C → T transitions, as observed in existing work [17].

**Fig 6:**
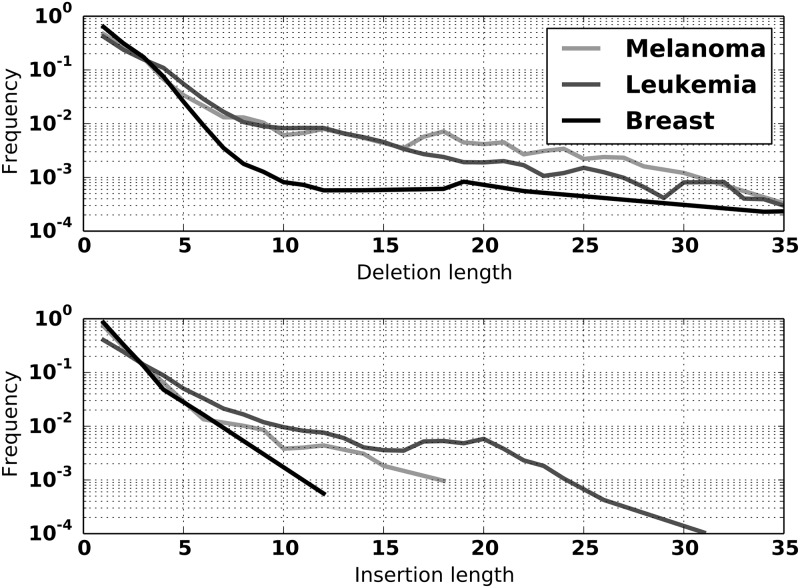
Insertion and deletion length distributions for Breast, Leukemia, and Melanoma models.

To help users get started with the simulator, we applied our mutation model generating scripts to the VCF files from the Genome In A Bottle consortium (GIAB) [12] for the sample NA12878, VCFs from the 1000 genomes project [13] and pooled variants from the International Cancer Genome Consortium (ICGC) simple somatic mutation files (SSM). From these data we compute frequency matrices for SNPs, indels, and structural variants. All variants are considered in the context of their surrounding sequence. Specifically, the script creates an index of the reference to capture the distribution of all trinucleotides in it. Then it locates each input variant on the reference, and reports the nucleotides one base before and one base after the variant, which comprises a *trinucleotide context* for each mutation (Figs 3–5). The frequency is calculated by finding the total number of instances of each trinucleotide transition, and dividing by the abundance of the original trinucleotide on the reference (for 1000 genomes data) or the germline trinucleotide (for ICGC). When heterozygous alleles are encountered, we randomly pick one, for simplicity. Small indel length distributions are also recorded (Fig 6).

We have generated default models for the NEAT toolkit for breast cancer, melanoma and leukemia using SSMs from the ICGC Release 20 TCGA projects BRCA-US and SKCM-US, and Release 20 of CLLE-ES [14]. Variant data from ICGC were pooled for all individuals with the same type of cancer.

We also provide a sample BED file for mutation rates in exons, introns and intergenic regions. The data were drawn from a GENCODE GRCh37 release 24 [15], dbSNP GRCh37.p13 build 146 [16] and several cancers as described in the previous section.

**Fig 7:**
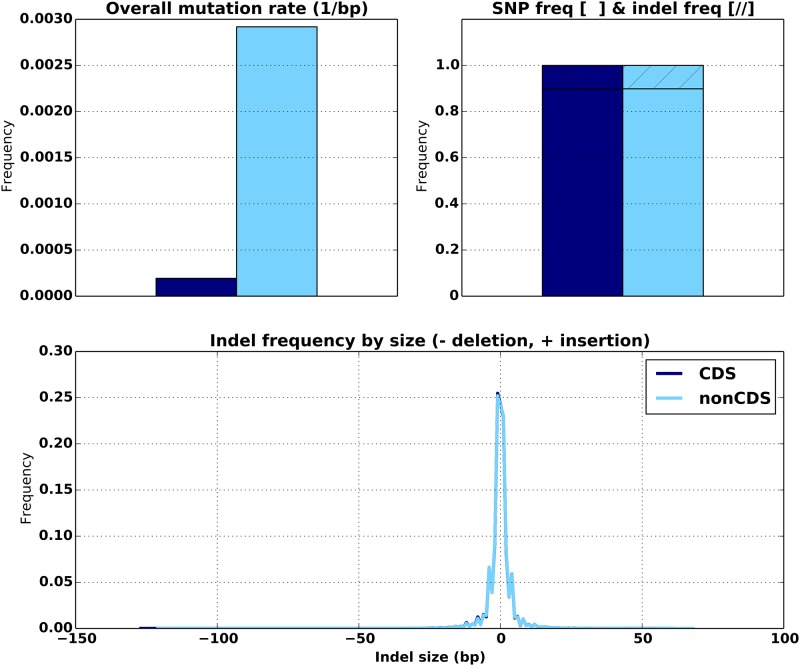
Comparison of mutation statistics between CDS (blue) and nonCDS (cyan) regions.

**Fig 8:**
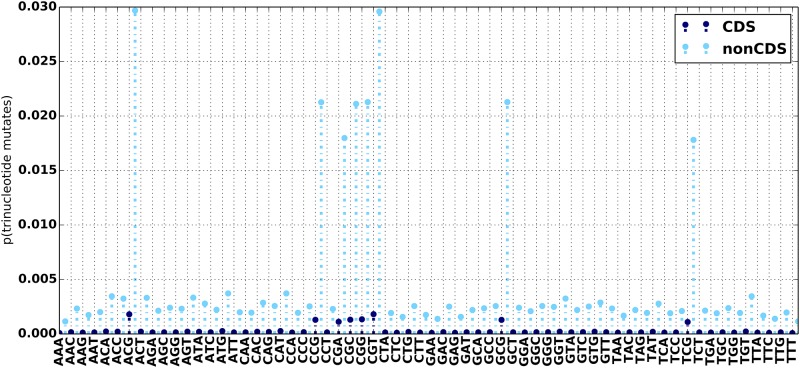
Trinucleotide mutation frequencies for NA12878 high confidence variants in CDS (blue) and nonCDS (cyan) regions.

Using the the set of high confidence calls made on NA12878 by GIAB, we confirmed that mutation statistics significantly differ in coding (CDS) and noncoding (nonCDS) regions of the genome (Fig 7). As expected we examine a much higher mutation rate in nonCDS regions. The trinucleotide mutation bias between CDS and nonCDS regions exhibit similar peaks, with nonCDS regions again having higher mutation rates (Fig 8). In contrast, both the SNP/indel fraction and the distributions of indel lengths in CDS and nonCDS appear identical (Fig 7). If these differences are important for the user, the list of CDS/nonCDS regions can be supplied via an input BED file in order to distinguish the overall mutation rates between them. The ability to supply two different mutation models, for CDS and nonCDS, into a single invocation of NEAT is part of our future code development effort. At present this can be worked around by generating multiple datasets with NEAT using separate mutation models and then merging the results with provided scripts.

### Sequencing Model Description

To emulate multiple sequencing platforms, NEAT derives a sequencing model from real FASTQ and BAM data. Similar to the mutation model, the *sequencing model* contains discrete distributions that are sampled by NEAT during read generation. The sequencing model contains three sub-models: *Quality score sub-model*, *sequencing error sub-model*, and *read sampling sub-model*.

**Quality Score Sub-model:** The quality score model contains distributions used to generate quality score strings for each synthetic read. The frequency of observed quality score transitions for each position along a read is obtained from an example FASTQ file. These frequencies are embedded in the transition matrices of a time-inhomogeneous Markov model, similarly to the methods utilized in existing simulators, such as MAQ [18] and pIRS. This yields many distributions of the form:

$$\begin{array}{l} P(\text{next quality score}=Q|\text{previous quality score}=Q^{\prime },\text{position}=P),\\ \text{for }P=1,\ldots ,L\text{ and }Q,Q^{\prime }=1,\ldots ,q_{max} \end{array}$$

Where *L* is the read length, and *q*_*max*_ is the highest quality score (e.g *q*_*max*_ = 41 for Phred+33 encoding). NEAT supports the use of separate models for forward and reverse reads when simulating paired-end datasets. These models allows us to estimate the dependence of quality scores on both the position within the read, and the previous base-call quality. By sampling quality score strings from this model, we can emulate the profiles of an input FASTQ file from arbitrary sequencing platforms.

**Sequencing Error Sub-model:** To mimic an observed distribution of sequencing errors, we process BAM files to compute sequencing substitution error base transition frequencies and sequencing indel error frequencies. The occurrence of sequencing errors is determined by selecting mismatched positions that are below a threshold quality score, as well as below a threshold variant allele frequency, and are not detected to be part of a larger event. These observed errors are used to estimate the following distributions that comprise the model:

1. *P*(error occurs ∣ quality score = *Q*)
2. *P*(substitution ∣ error occurs), *P*(insertion ∣ error occurs), *P*(deletion ∣ error occurs)
3. *P*(substituted base = *X* ∣ current base = *Y*, substitution error occurs)
4. *P*(indel length ∣ indel error occurs)
5. *P*(inserted base = *X* ∣ insertion error occurs)

Errors are inserted into the read data as follows. For each position in the read we insert an error with probability proportional to the quality score at that position. By sampling from the distributions (b) as given above, we determine whether the error should be a substitution, insertion, or deletion. If the error is determined to be a substitution, we sample from the distribution (c) to determine what new base should replace the nucleotide at the error position. If the error is determined to be an insertion or deletion, we sample from distribution (d) to determine its length. Finally, if the error is an insertion, we successively sample from (e) to create the new erroneous sequence of nucleotides that will be inserted into the read.

To help users create their own sequencing error sub-models, we provide a script that processes alignments to derive the statistics described in the previous section. A position within a read is determined to be a sequencing error if it meets all of the following conditions:

- The position contains a spurious mismatch or indel (up to a specified length) that has low variant allele frequency (i.e. is not supported by other reads)
- The position in the supporting read is below a specified quality threshold
- The mapping quality of the supporting read is above a specified threshold

**Fig 9:**
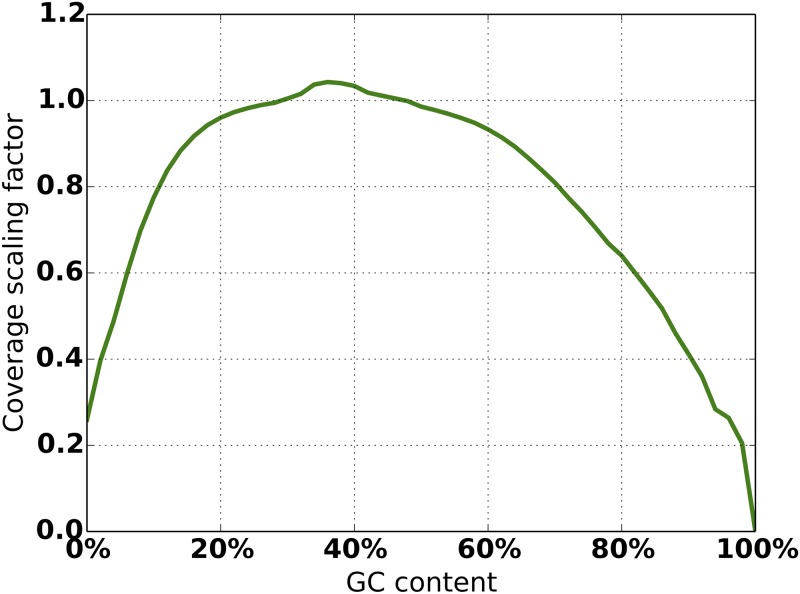
Empirical GC% coverage bias from an example BAM file.

**Fig 10:**
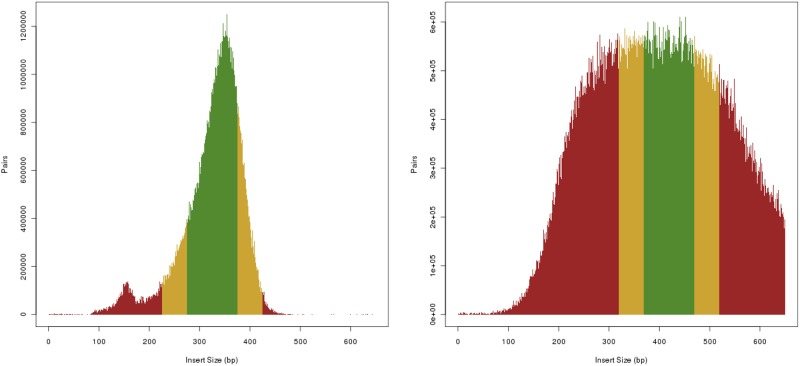
Empirical insert size distribution from two example BAM files. (Left) ICGC donor DO35138: dcc.icgc.org/donors/DO35138, (Right) ICGC donor DO221544: dcc.icgc.org/donors/DO221544, both from project PACA-CA.

**Read Sampling Sub-model:** In addition to sequencing error statistics, the input BAM file is used to compute GC% coverage bias and paired-end fragment length distributions. Using the BEDTools genomecov tool [19], GC% coverage bias is computed by sliding non-overlapping windows of a fixed size along the generated track and binning the coverage value by local GC content. The counts are normalized by the average coverage of the entire alignment to yield multipliers to scale coverage during simulation (Fig 9). Paired-end fragment length distribution is computed using the template-length field in the alignment data (Fig 10).

### Workflow Evaluation Tools

The simulator includes a set of scripts to process BAM and VCF workflow output to determine alignment and variant detection accuracies. The performance of an aligner can be assessed by manually comparing the golden alignment to the BAM produced by the aligner. Or more simply, by comparing the mapped position and CIGAR string to the values embedded in the read names of the synthetic data using an included script. Similar analyses can be performed with other tools, such as LAVEnder [20] (In development at the time of this writing), which can identify and plot multiple types of alignment errors.

**Fig 11:**
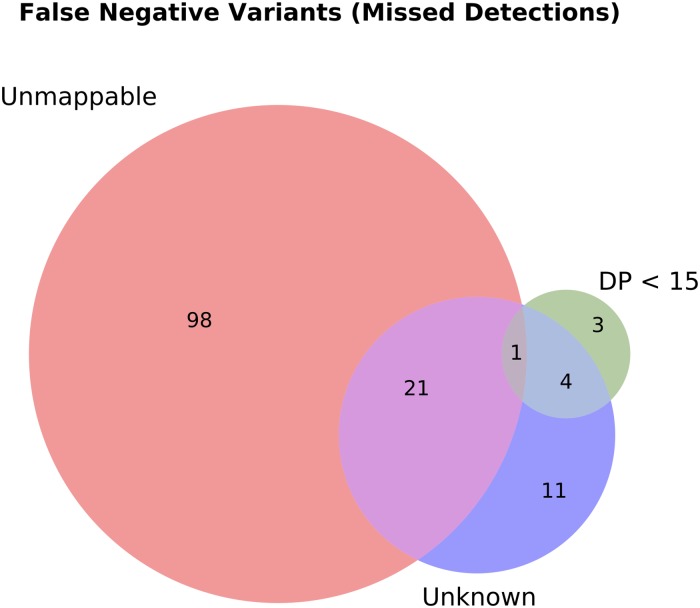
Example false negative variant call diagnosis for a toy dataset: Several hundred variants were introduced into a 10M subset of human chromosome 21. The false negative variants were those that were inserted into the data by NEAT, but were not recovered by a particular variant calling workflow (Novoalign → Haplotype Caller, following GATK best practices). In this example we see that a majority of the false negatives were due to variants having been inserted into regions that were not uniquely mappable with the simulated read lengths. A lower number of false negatives were due to inadequate coverage (DP).

NEAT includes a VCF comparison script to compare workflow output to the golden VCF containing inserted variants. The comparison is similar to vcf-compare (part of the vcftools suite [21]), with the added advantage of using coverage and mappability information to facilitate manual investigation of false positive (FP) and false negative (FN) variant calls. When comparing a VCF produced by a workflow to the golden VCF produced by NEAT, our comparison script will count FP and FN variants and split them into separate output files for manual inspection. Because the VCF representation of a mutation (position, reference allele → alternate allele) is not unique, our script has an optional feature to detect variants (or groups of variants) that are equivalent but not represented identically between the input VCF files. Additionally, our comparison script offers the ability to diagnose FN variant calls by counting how many of them originated from positions that were either not well covered in the golden alignment or were from unmappable regions of the reference sequence (Fig 11).

## Discussion

As described in previous sections, the NEAT read simulator is an amalgam of features present across a variety of software packages (Table 1) with additional consideration paid to deriving models from real data. By using this approach, we can generate NGS datasets useful for a wider variety of applications, where sequencing and mutation characteristics could vary considerably.

### NEAT use cases

Many of NEAT’s features were motivated by the variety of needs to satisfy many use cases.

The classic use of synthetic read simulators is for evaluating alignment and variant calling software, especially in tough cases, such as variants present in genomic regions that are difficult to map due to their repetitiveness. Simulation permits insertion of variants that would not have been present in a regular VCF.

Simulated data can be used to determine optimal sequencing properties to compensate for the current shortcomings in variant calling. For example, a number of datasets can be built on the same mutation model by varying read lengths, coverage, fragment length, etc., in an effort to study the effect of these parameters on the ease of variant calling and downstream analyses. Allowing for empirical coverage bias and fragment lengths was motivated specifically by our observation that in real data these distributions are not well characterized by a simple Gaussian (Figs 9 and 10).

NEAT can be used to simulate sequencing experiments from any organism, including human, other mammals, plants, even heterogeneous populations. The scripts for extracting mutation and sequencing models are agnostic of species, while the simulator itself allows arbitrary ploidy setting. Polyploid genome simulation can be useful for crop plants, such as sugarcane [22], wheat [23] and soybean [24] It is unfortunately difficult to construct a good quality genome assembly in a highly polyploid species, and one might therefore argue against trying to simulate sequencing experiments based on a faulty reference. We suggest a different viewpoint: one could experiment with various hypothetical references in simulation, and thus reconstruct the correct genome assembly by comparing the simulated reads with real sequencing data. Ploidy can also be used as a proxy to simulate a distribution of haplotypes in a mixed population, such as a heterogeneous tumor sample.

We purposely designed the simulator to allow for arbitrary mutation models in order to address the heterogeneity of mutation characteristics across different groups of cancers (Figs 3–5). This ability to faithfully reproduce a mutational profile is useful for training physicians, genetic counselors and analysts in interpretation of the results of variant calling procedures.

### NEAT limitations, in comparison to other simulators

Several features of NEAT were inspired by existing tools, but have not been as comprehensively implemented. Wessim’s “probe hybridization” approach is useful for simulating whole exome sequencing, without a current NEAT equivalent. NEAT simply samples reads that cover targeted regions with an increased frequency proportional to the on-target/off-target coverage ratio adjustable by user (defaulted to 98%/2% of the average coverage). This is akin to the “ideal target” functionality of Wessim, and is likewise unrealistic.

Our approach to quality score profiles is similar to Markov model approaches and pIRS (and earlier, MAQ), however we assume the quality score accurately represents the probability of erroneous base calls.

Additionally, pIRS adjusts the likelihood of substitution vs. indel sequencing errors as a function of position along a read, learned from data. NEAT considers this likelihood to be uniform throughout. While the goal of NEAT is not to exhaustively emulate all peculiarities of all sequencing technologies, comprehensive models in existing tools serve to guide the development of new features in future versions of the software, outlined below in the section Future Directions.

Finally, NEAT is a toolkit that contains software for deriving mutation and sequencing models, simulating sequencing data, and evaluating alignment and variant calling algorithms. In this regard NEAT could be easily confused with VarSim [10], itself a powerful computational framework with similar functions. However, VarSim is a wrapper around other simulators, and thus inherits all their features and limitations. Indeed, VarSim could use NEAT gen-reads function much like it uses DWGSIM and ART. VarSim is human-centric, hypothesizes diploid genomes and uses human variant databases to perturb the reference prior to simulation. It has excellent advanced features specifically for human cancer, such as automatic generation of structural variants, simulation of germline and somatic genomes, and mixtures of reads from both. In contrast, NEAT is species-agnostic and features arbitrary, user-defined ploidy. NEAT also allows more control over the mutation models in terms of relative abundance of different variant kinds, their qualities and spatial distributions. NEAT is easier to install and use, because it is self-contained and requires only one command for execution.

### Realism of inserted variants

Our simulator can introduce mutations in two ways: deterministically from an input VCF supplied by the user, and stochastically according to the supplied mutation models. Both mechanisms can be used to improve the realism of inserted variants, but also carry over the drawbacks of the variant calling procedure used to identify the variants in the original dataset. Some variants are difficult to call, either because of their location in a repetitive region that is difficult to map [25], or due to the complex nature of the variant itself. Those hard-to-call variants will be under-represented in the datasets from which the mutation models are constructed, and also in the simulated reads. NEAT has several user options to remedy this situation. First, an input BED file enables users to explicitly set the mutation rate per-region, allowing users to take extra care in handling those hard-to-map regions, if it is important for their simulation experiment. Second, in the absence of such a BED file, the properly set background mutation probability ensures that *all* regions are subject to that average mutation rate uniformly across the genome. This option can be of value when using the simulated data to test aligners and variant callers. On the other hand, when training physicians and analysts using known variants of any given cancer, it is useful to faithfully reproduce mutations *as we know them today*, as opposed to guessing what may have been missed. The stochastic mutation models based on previously called variants in different cancers should be sufficient in that use case.

The ability to set mutation rates per region via an input BED file is particularly useful when the experiment is sensitive to the differences between exons, introns, coding sequences, and other regions. It is critical for the user to have control over these values, as one setting does not apply to all situations [26–28]. Because NEAT samples reads from reference regions in a sliding-window fashion (as to not require storing the entire reference sequence in memory), position-specific mutation rates affect the windows overlapping the provided coordinates. This has the effect of slightly smoothing out any abrupt differences in the mutation rates between adjacent regions. Mutation probabilities tend not to change abruptly along the genome at that scale [29]. The smoothing effect can be controlled, to some extent, by adjusting the length of the BED regions, but the window size used internally by NEAT sets the lower limit on the BED region length within which the mutation rate can be distinguishable.

If smaller-scale effects, such as around CpG islands [26, 30], are important to the user, they should be included in the input VCF file: NEAT inserts those variants verbatim. In addition, despite using pooled genotype data, NEAT does not purposefully simulate events such as linkage disequilibrium or other population-level genetics information. To simulate this type of data, known variants can be introduced using the VCF file, or tools such as HAPGEN2 [31] can be used to simulate disease SNPs that can be subsequently introduced into NEAT reads.

### NEAT Validation

To validate the output of NEAT, we performed several simulation workflows to confirm that the mutation distributions and sequencing characteristics of simulated data match the derived mutation and sequencing models of real data, respectively (S1 File).

We validated NEAT’s ability to generate synthetic mutations using a mutation model derived from simple somatic mutations in the breast cancer data from ICGC described in the previous sections. We found no appreciable difference between the mutation models constructed on the raw variants and those produced by NEAT, indicating that the stochastic properties of mutations present in that dataset were preserved in simulation.

Similarly, we computed sequence and alignment statistics on data generated by NEAT using FastQC [32] and BAMQC (an in-house script that measures basic alignment statistics such as insert size distributions and sequencing error positions). We found that a majority of the figures produced by these tools were nearly identical, in particular the per-base sequence quality, per-sequence GC content and the insert size distributions. A few metrics computed from the synthetic data do appear to have idealized shapes due to a number of sequencing nuances that we do not emulate, such as the presence of adapter sequences and heavily duplicated sequences.

### Future directions

**Augmented mutation models:** In ongoing work we are adding more scripts and user options for greater control over the parameters of the simulated datasets. These options include more detailed mutation models, such as insertion motifs, heterozygous/homozygous ratios, and distinct mutation probabilities for synonymous vs. nonsynonymous mutations [33] and mutations occurring in coding vs noncoding regions of the genome. We will also allow the insertion of randomly generated large structural variants. Users will be able to specify ploidy as a function of coordinate on the reference, via BED file, which will be useful for simulating copy number variation.

**Improved sequencing models:** Additionally, we are augmenting the sequencing models to accommodate generating FASTQ data with varying read lengths, appropriate for long read sequencing technologies, such as the PacBio RS II and Illumina Moleculo.

Other targeted sequencing experiments, such as ChIP-seq or RNA-seq, can theoretically be simulated via the same methods as for exome sequencing simulation, i.e. by supplying a BED file with sequencing targets (such as potential protein binding sites for ChIP-seq). However, these data show highly variable coverage among the targeted regions, and this cannot currently be simulated by NEAT. Our next step is introducing into NEAT a capability to retrieve coverage information from the input BED file individually on a per region basis, which will dramatically improve the realism of ChIP-seq and RNA-seq simulation.

**Simulating genomic lineages:** We are also developing wrappers around NEAT to generate combinations of reads representative of populations of individuals. The reference will be progressively mutated by applying the supplied mutation models in series, while keeping track of the exact mutations introduced. This feature will be particularly useful for modeling cancer cell populations. The tumor sample clonality will be simulated by selecting the appropriate members of the modeled lineage, simulating their sequencing data from respective mutated references, and mixing those in proportions that correspond to the clonality levels.

## Conclusions

We have developed NEAT, a highly flexible simulator for generating synthetic FASTQ, BAM, and VCF files. It is capable of emulating the characteristics of various sequencing platforms by learning sequencing models directly from real-world FASTQ and BAM data. It can simulate whole-genome data of different populations by learning mutation models directly from variant call data. Additionally, NEAT supports targeted sequencing (e.g. whole exome) via an input BED file. We have used NEAT to estimate mutation statistics of a population of individuals with breast cancer, and have showcased the ability to create datasets with a wide range of mutation and sequencing error characteristics.

Improving the quality of simulated data has many benefits. Simulated data with fully known true positives can be used for developing new algorithms, testing the bounds of existing software and fairly comparing different software to each other. It can be used for teaching, allowing educators to generate real-looking datasets for students to learn on, and for research, by testing hypotheses about reference genome organization.

NEAT provides the means to generate standard data against which diagnostic software packages can be assessed, and thus estimates of false positive and false negative rates can be quantified. The rapid creation of realistic simulated datasets in this way can be used as an internal control by which software pipelines can self-test, optimize parameters, and uncover the capabilities and limitations of computational analyses and sequencing technologies. Ultimately, the availability of datasets such as these are needed to provide statistical confidence in genomic diagnostics, in order for applications of genomic analysis software gain widespread approval and adoption.

## Supporting Information

S1 File NEAT Validation. This document contains the results of workflows designed to assess the validity of the synthetic data produced by NEAT.
(PDF)

## References

[1] Shanks N, Greek R, Greek J. Are animal models predictive for humans? Philosophy, Ethics, and Humanities in Medicine. PEHM. 2009;4:2 10.1186/1747-5341-4-2 19146696 PMC2642860

[2] Huang W, Li L, Myers JR, Marth GT. ART: a next-generation sequencing read simulator. Bioinformatics. 2012;28(4):593–594. 10.1093/bioinformatics/btr708 22199392 PMC3278762

[3] Caboche S, Audebert C, Lemoine Y, Hot D. Comparison of mapping algorithms used in high-throughput sequencing: application to Ion Torrent data. BMC genomics. 2014;15(1):264 10.1186/1471-2164-15-264 24708189 PMC4051166

[4] McElroy KE, Luciani F, Thomas T. GemSIM: general, error-model based simulator of next-generation sequencing data. BMC genomics. 2012;13(1):1 10.1186/1471-2164-13-74 22336055 PMC3305602

[5] Hu X, Yuan J, Shi Y, Lu J, Liu B, Li Z, et al pIRS: Profile-based Illumina pair-end reads simulator. Bioinformatics. 2012;28(11):1533–1535. 10.1093/bioinformatics/bts187 22508794

[6] Pattnaik S, Gupta S, Rao AA, Panda B. SInC: an accurate and fast error-model based simulator for SNPs, Indels and CNVs coupled with a read generator for short-read sequence data. BMC bioinformatics. 2014;15(1):1 10.1186/1471-2105-15-40 24495296 PMC3926339

[7] Whole Genome Simulator for Next-Generation Sequencing;. Accessed: 2016-02-01. http://github.com/nh13/dwgsim.

[8] Kim S, Jeong K, Bafna V. Wessim: a whole-exome sequencing simulator based on in silico exome capture. Bioinformatics. 2013;p. btt074 10.1093/bioinformatics/btt074 23413434 PMC3624799

[9] Holtgrewe M. Mason–a read simulator for second generation sequencing data. Technical report FU Berlin. 2010;.

[10] Mu JC, Mohiyuddin M, Li J, Bani Asad N, Gerstein MB, Abyzov A, Wong WH, Lam HYK. VarSim: a high-fidelity simulation and validation framework for high-throughput genome sequencing with cancer applications. Bioinformatics. 2015; 31(9):1469–1471. 10.1093/bioinformatics/btu828 25524895 PMC4410653

[11] van der Walt S, Colbert SC, Varoquaux G. The NumPy Array: A Structure for Efficient Numerical Computation. Computing in Science & Engineering. 2011; 13:22–30. 10.1109/MCSE.2011.37

[12] Zook JM, Chapman B, Wang J, Mittelman D, Hofmann O, Hide W, Salit M. Integrating human sequence data sets provides a resource of benchmark SNP and indel genotype calls. Nature Biotechnology. 2014; 32:246–251 10.1038/nbt.2835 24531798

[13] The 1000 Genomes Project Consortium A global reference for human genetic variation. Nature. 2015; 526:68–74. 10.1038/nature15393 26432245 PMC4750478

[14] Puente XS, Beà S, Valdés-Mas R, Villamor N, Gutiérrez-Abril J, et al Non-coding recurrent mutations in chronic lymphocytic leukaemia. Nature. 2015;526:519–524. 10.1038/nature14666 26200345

[15] Harrow J, Frankish A, Gonzalez JM, Tapanari E, Diekhans M, et al GENCODE: The reference human genome annotation for The ENCODE Project. Genome Research. 2012;22:1760–1774. 10.1101/gr.135350.111 22955987 PMC3431492

[16] Bethesda (MD): National Center for Biotechnology Information, National Library of Medicine. Database of Single Nucleotide Polymorphisms (dbSNP). dbSNP Build ID: 146. Available from: http://www.ncbi.nlm.nih.gov/SNP/

[17] Hodis E, Watson IR, Kryukov GV, Arold ST, Imielinski M, Theurillat JP, et al A landscape of driver mutations in melanoma. Cell. 2012;150(2):251–263. 10.1016/j.cell.2012.06.024 22817889 PMC3600117

[18] Li H, Ruan J, Durbin R. Mapping short DNA sequencing reads and calling variants using mapping quality scores. Genome Research. 2008;18(11):1851–1858. 10.1101/gr.078212.108 18714091 PMC2577856

[19] Quinlan AR, Hall IM. BEDTools: a flexible suite of utilities for comparing genomic features. Bioinformatics. 2010;26(6):841–842. 10.1093/bioinformatics/btq033 20110278 PMC2832824

[20] Břinda K, Boeva V, Kucherov G. RNF: a general framework to evaluate NGS read mappers. Bioinformatics. 2016;32(1):136–139. 10.1093/bioinformatics/btv524 26353839 PMC4681991

[21] Danecek P, Auton A, Abecasis G, Albers CA, Banks E, DePristo MA, et al The variant call format and VCFtools. Bioinformatics. 2011;27(15):2156–2158. 10.1093/bioinformatics/btr330 21653522 PMC3137218

[22] Premachandran MN, Prathima PT, Lekshmi M. SUGARCANE AND POLYPLOIDY—A REVIEW. Journal of Sugarcane Research 2011;1(2):1–15.

[23] Isidore E, Scherrer B, Chalhoub B, Feuillet C and Keller B. Ancient haplotypes resulting from extensive molecular rearrangements in the wheat A genome have been maintained in species of three different ploidy levels. Genome Research 2007;15:526–536. 10.1101/gr.3131005 PMC1074367 15805493

[24] Schlueter JA, Lin J-Y, Schlueter SD, Vasylenko-Sanders IF, Deshpande S, Yi J, O’Bleness M, Roe BA, Nelson RT, Scheffler BE, Jackson SA and ShoemakerEmail RC. Gene duplication and paleopolyploidy in soybean and the implications for whole genome sequencing. BMC Genomics 2007;8:330 10.1186/1471-2164-8-330 17880721 PMC2077340

[25] Treangen TJ, Salzberg SL. Repetitive, DNA and next-generation sequencing: computational challenges and solutions. Nature Reviews Genetics 2013;13(1):36–46. 10.1038/nrg3117 22124482 PMC3324860

[26] Hodgkinson A, Eyre-Walker A. Variation in the mutation rate across mammalian genomes. Nature Reviews Genetics 2011;12:756–766. 10.1038/nrg3098 21969038

[27] Olivier M, Hollstein M, Hainaut P. TP53 mutations in human cancers: origins, consequences, and clinical use. Cold Spring Harbor Perspective in Biology 2010;2:a001008 10.1101/cshperspect.a001008 20182602 PMC2827900

[28] Polak P, Lawrence MS, Haugen E, Stoletzki N, Stojanov P, Thurman RE, Garraway LA, Mirkin S, Getz G, Stamatoyannopoulos JA, Sunyaev SR. Reduced local mutation density in regulatory DNA of cancer genomes is linked to DNA repair. Nature Biotechnology 2014;32(1):71–75. 10.1038/nbt.2778 24336318 PMC4116484

[29] Gaffney DJ, Keightley PD. The scale of mutational variation in the murid genome. Genome Research 2005;15:186–1094. 10.1101/gr.3895005 PMC1182221 16024822

[30] Bird AP. CpG-rich islands and the function of DNA methylation. Nature 1986;321:209–213. 10.1038/321209a0 2423876

[31] Su Z, Marchini J, Donnelly P. HAPGEN2: simulation of multiple disease SNPs. 2011;27(16):2304–2305. 10.1093/bioinformatics/btr341 21653516 PMC3150040

[32] Andrews S. FastQC: A quality control tool for high throughput sequence data. Cambridge, UK: Babraham Institute (2011)

[33] Subramanian S, Kumar S. Neutral substitutions occur at a faster rate in exons than in noncoding DNA in primate genomes. Genome Research. 2003;13:838–844. 10.1101/gr.1152803 12727904 PMC430942

