# Supplementary material for: Simulating Next-Generation Sequencing Datasets from Empirical Mutation and Sequencing Models
Source: PLoS One. 2016 Nov 28;11(11):e0167047. doi: 10.1371/journal.pone.0167047 (PMC5125660; doi:10.1371/journal.pone.0167047)
Supplement: S1 File — This document contains the results of workflows designed to assess the validity of the synthetic data produced by NEAT. (PDF) [file pone.0167047.s001.pdf]

Simulating Next-Generation Sequencing Datasets from Empirical Mutation and Sequencing Models

Supplementary materials

1) Mutation Model Validation

The purpose of this experiment is to validate that the mutations being inserted into the synthetic data are correctly being sampled from the distributions learned from real example data. In this experiment we compare the mutation statistics of an input database to the VCF produced by NEAT containing synthetic variation.

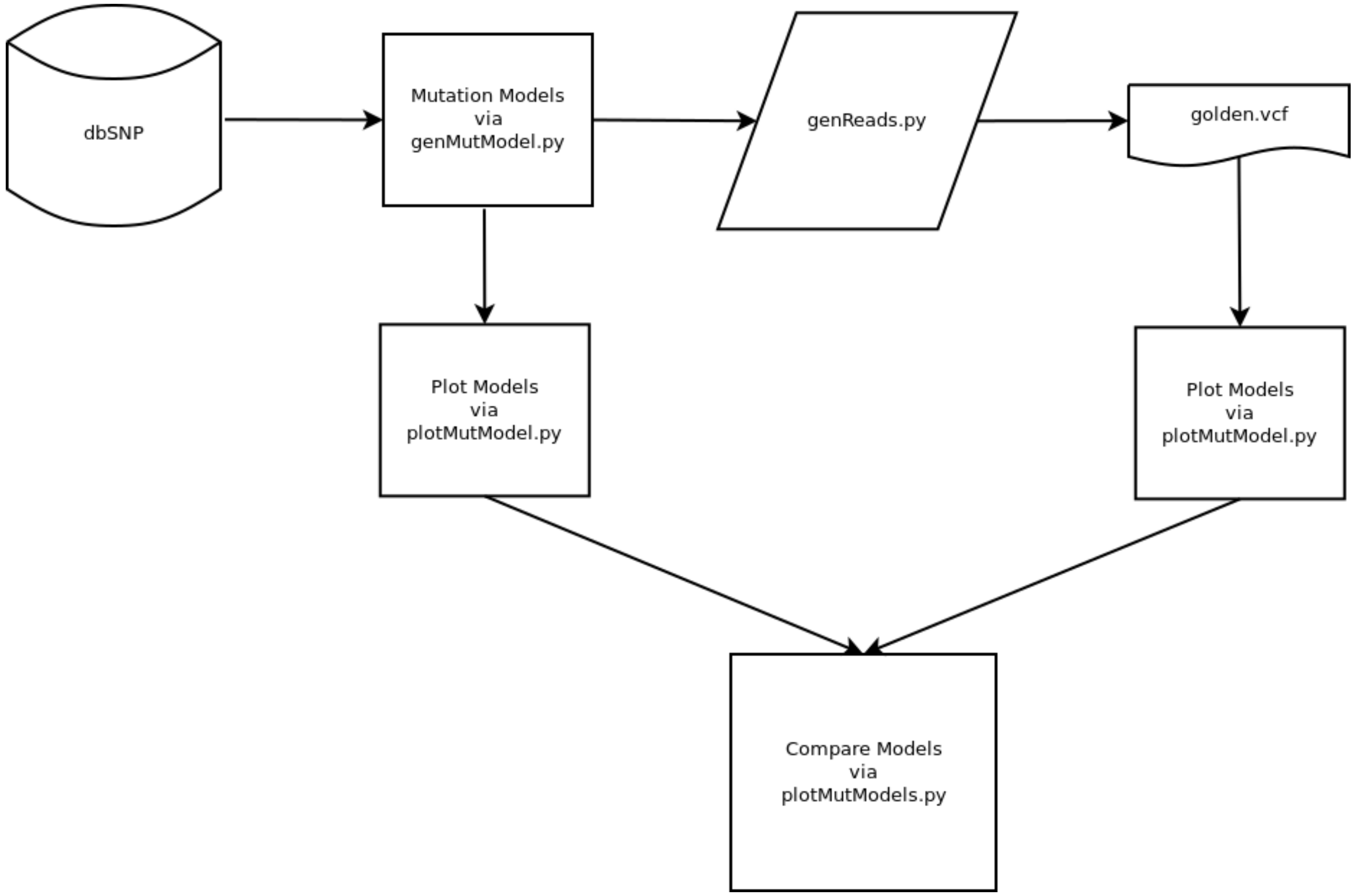

Supplementary Figure 1. Validation workflow for NEAT mutation evaluation. Mutation models are constructed based on a real variant set, and based on a synthetic VCF produced by NEAT. The two are compared to detect any differences in the overall mutation rate, the probability distribution of the indel lengths, trinucleotide likelihood priors and the trinucleotide transition probabilities.

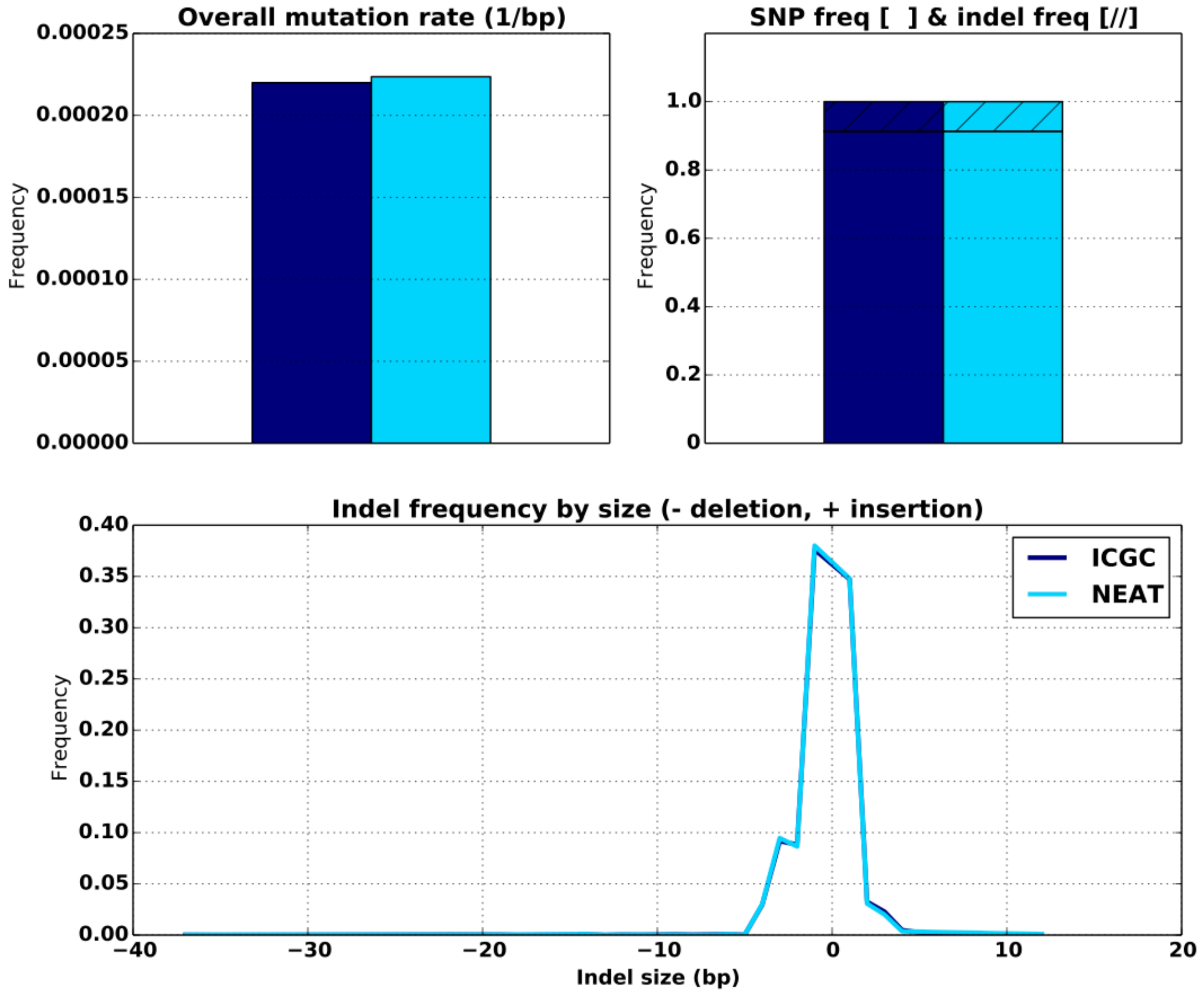

Supplementary Figure 2. Comparison of mutation statistics between ICGC dataset (blue) used to derive models, and synthetic data produced by NEAT (cyan). We note that the statistics are nearly identical for both overall mutation rate (top left), SNP/indel fraction (top right) and indel length distribution (bottom).

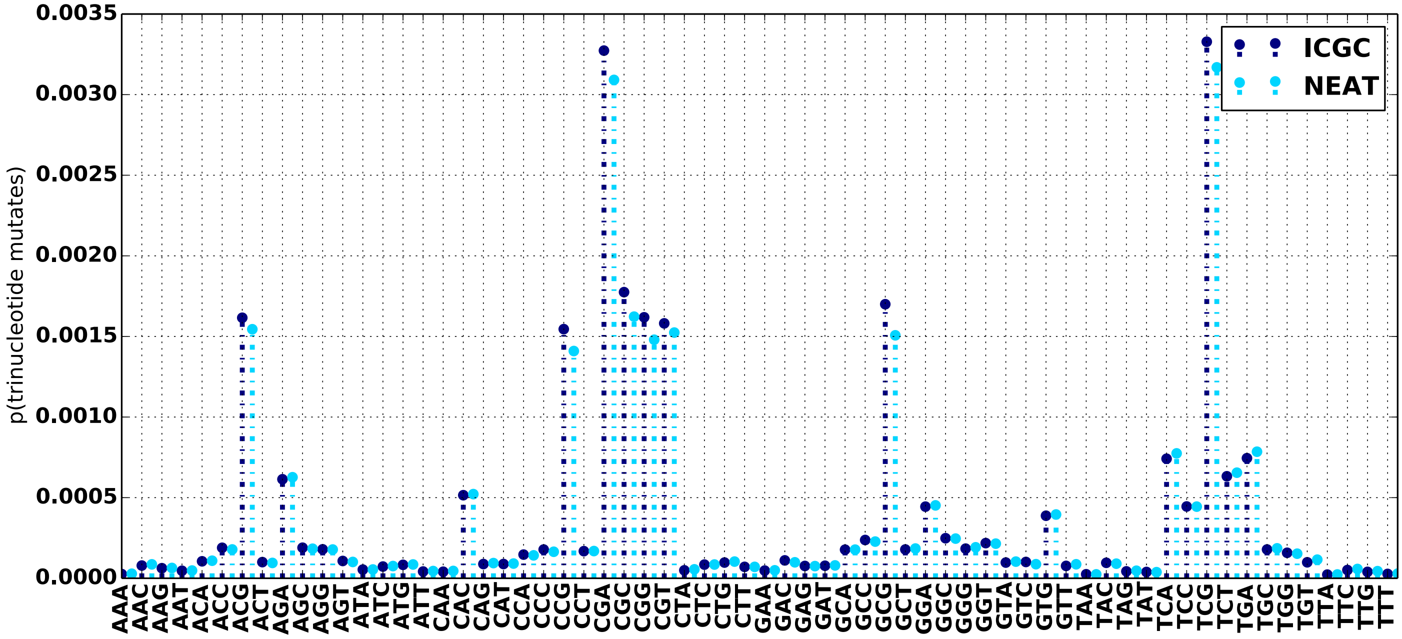

Supplementary Figure 3. The trinucleotide mutation frequencies of the ICGC (dark blue) dataset used to derive the mutation model match the mutation frequencies of the synthetic data generated by NEAT using the model (light blue).

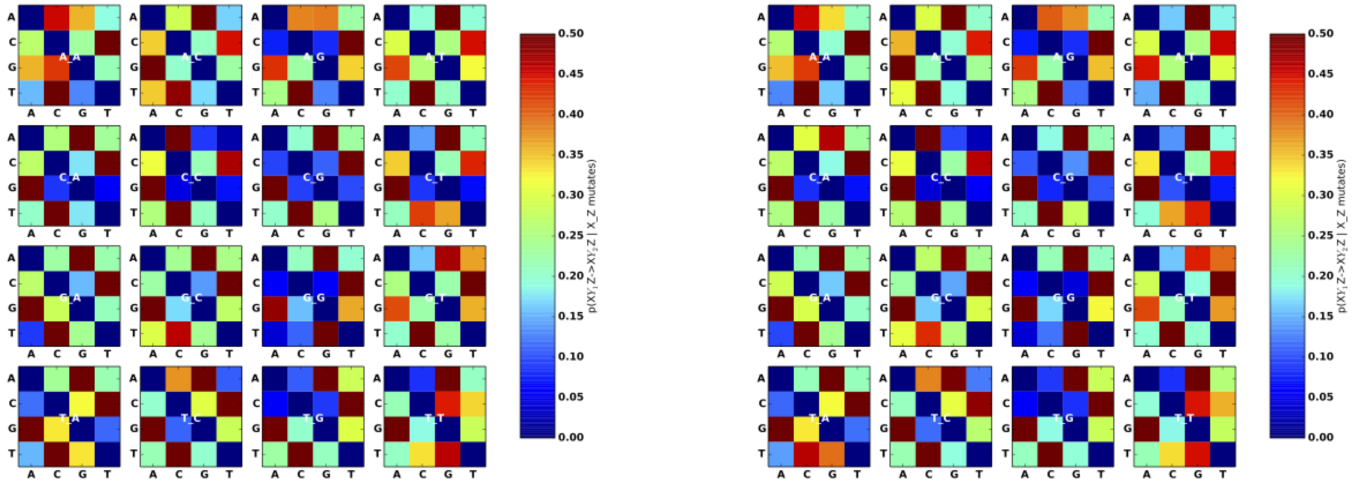

Supplementary Figure 4. The conditional trinucleotide mutation frequencies of the ICGC (dark blue) dataset used to derive the mutation model match the mutation frequencies of the synthetic data generated by NEAT using the model (light blue).

2) Sequencing Model Validation

The purpose of this experiment is to validate that the sequencing characteristics from NEAT simulated data is similar to the real data it is modeled after. In this experiment we compare the FASTQC and BAMQC (an in-house tool for computing alignment metrics) statistics computed from real and simulated datasets generated using models derived from the same real data. An overview of the workflow is given below:

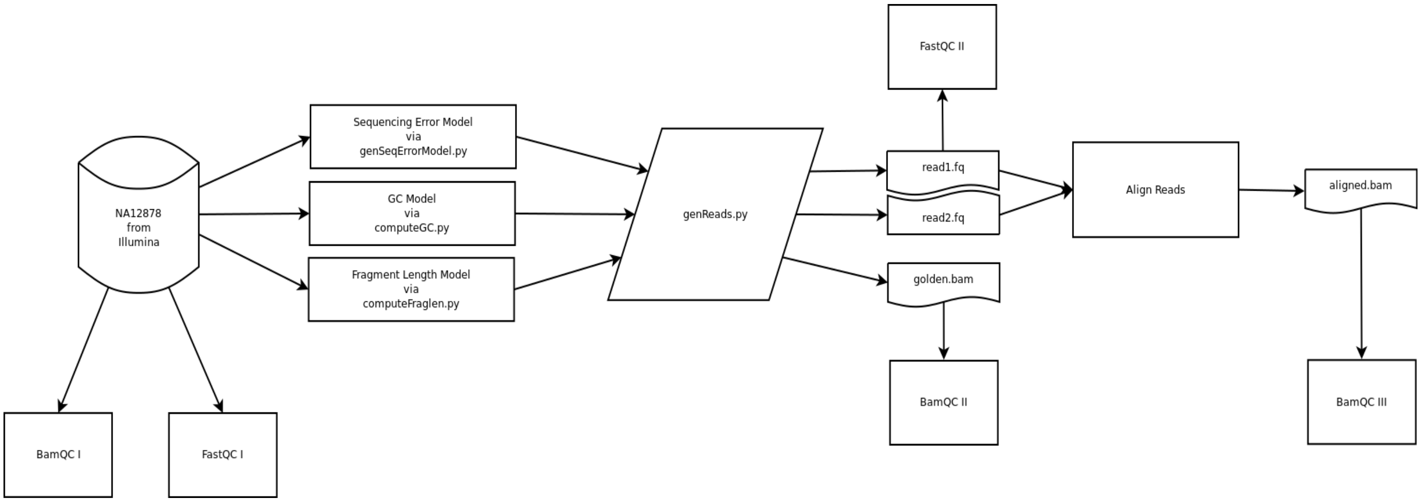

Supplementary Figure 5. Validation workflow for NEAT sequencing model evaluation.

From the FASTQC report, we see that in most metrics the simulated data is indistinguishable from the real dataset. We noted differences in per-sequence GC content and overrepresented kmers likely due to aspects of sequencing technology that we are not emulating.

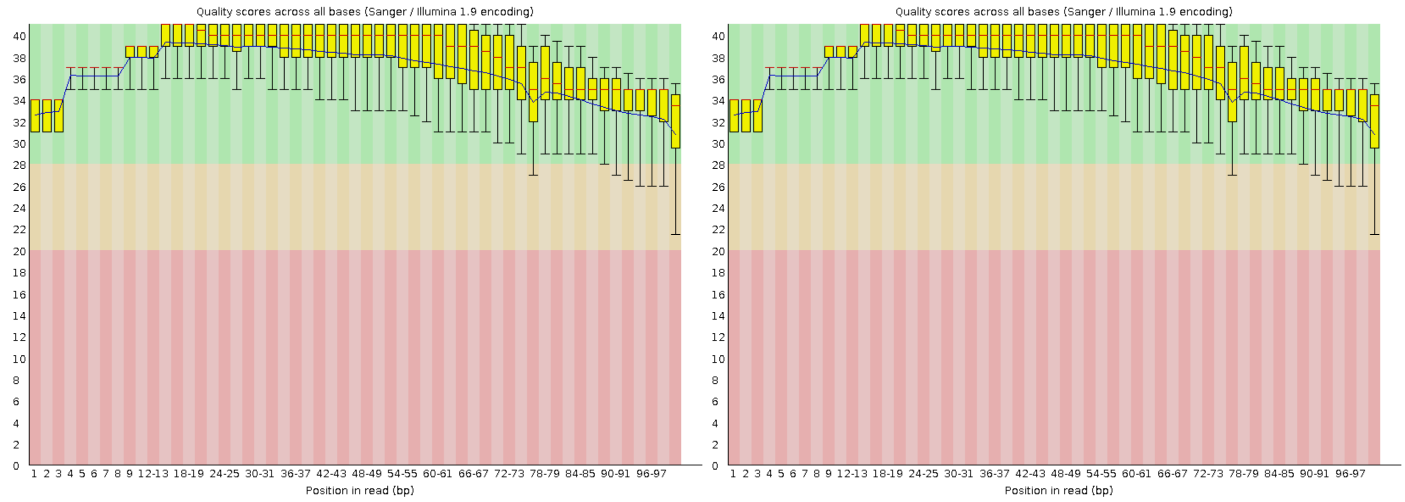

Supplementary Figure 6. Per-base sequence quality of real (left) and synthetic (right) datasets. The nearly identical distributions indicate that the quality strings sampled from the sequencing model have aggregate statistics highly similar to the quality string statistics of the data used to generate the model.

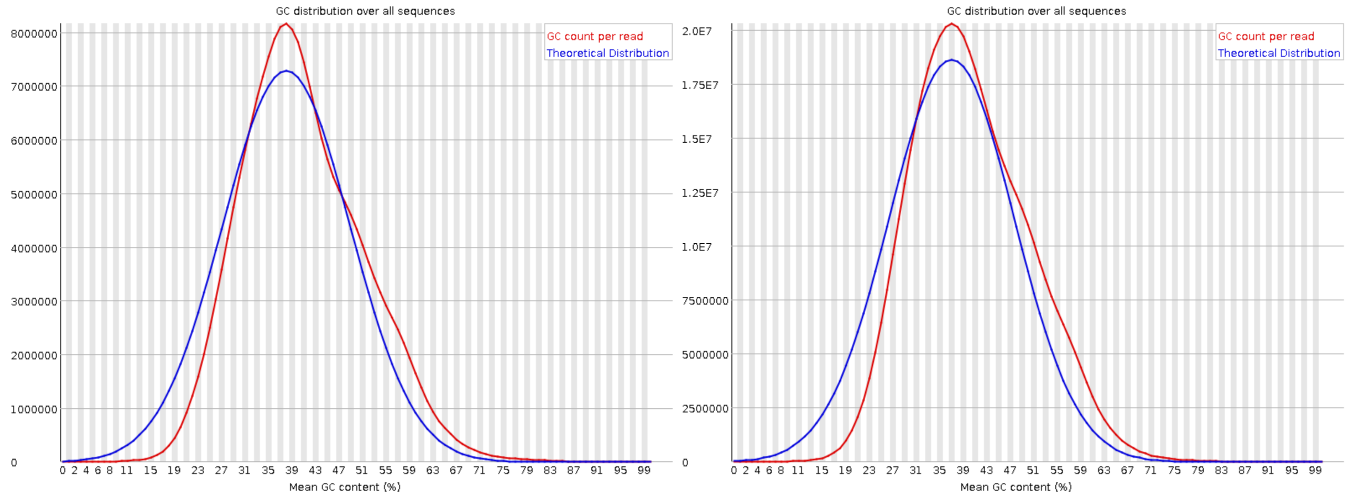

Supplementary Figure 7. Per-sequence GC content of real (left) and synthetic (right) datasets. The similar shape of the red curves suggests that GC bias is being simulated accurately.

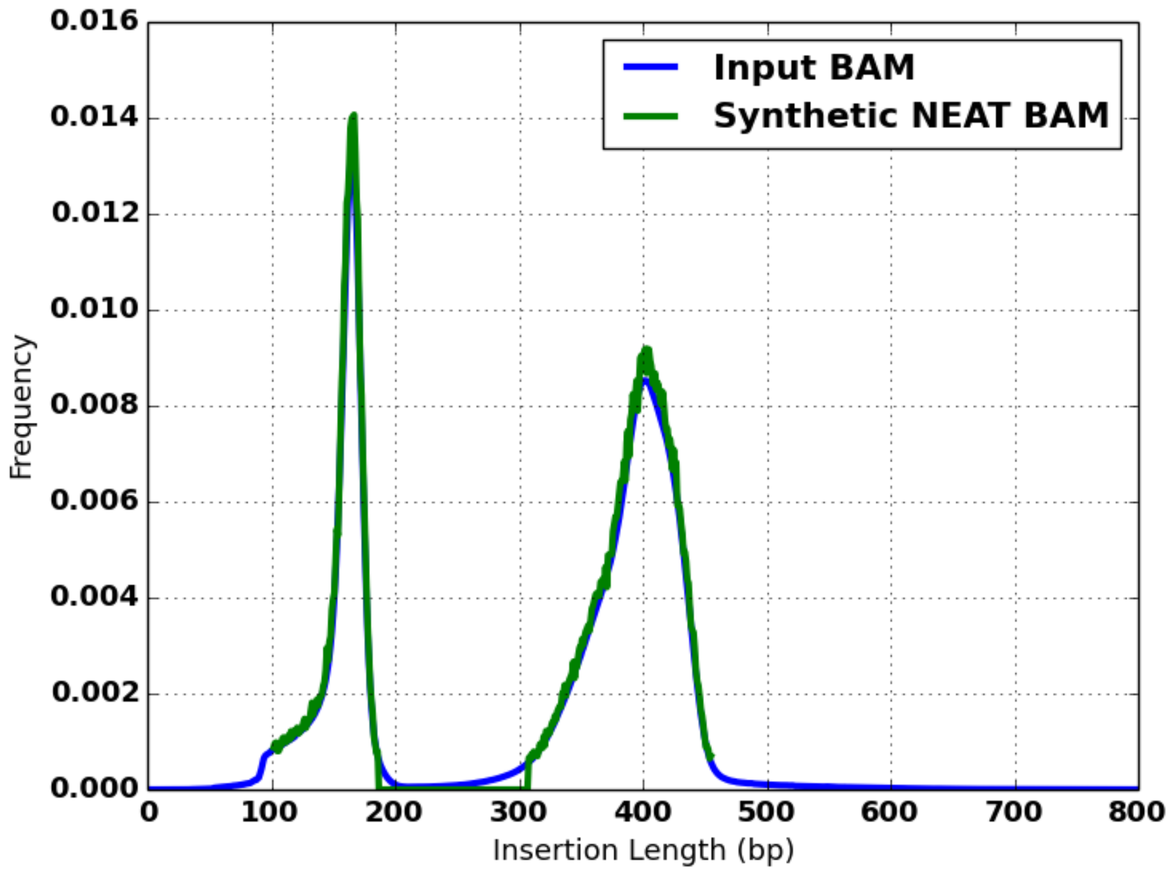

Supplementary Figure 8. Insert size distributions for real (blue) and synthetic (green) datasets. The shape of the distribution is nearly identical, indicating the model derived from real data is being accurately sampled from.
